# Exposure to Secondhand Smoke in Terraces and Other Outdoor Areas of Hospitality Venues in Eight European Countries

**DOI:** 10.1371/journal.pone.0042130

**Published:** 2012-08-01

**Authors:** Maria J. López, Esteve Fernández, Giuseppe Gorini, Hanns Moshammer, Kinga Polanska, Luke Clancy, Bertrand Dautzenberg, Agnes Delrieu, Giovanni Invernizzi, Glòria Muñoz, Jose Precioso, Ario Ruprecht, Peter Stansty, Wojciech Hanke, Manel Nebot

**Affiliations:** 1 Public Health Agency of Barcelona, Barcelona, Spain; 2 Consorcio de Investigación Biomédica en Red especializado en Epidemiología y Salud Pública (CIBERESP), Barcelona, Spain; 3 Institut d’Investigació Biomèdica Sant Pau, Barcelona, Spain; 4 Tobacco Control Unit, Cancer Control and Prevention Programme, Institut Català d’Oncologia (ICO), L’Hospitalet de Llobregat, Spain; 5 Cancer Control and Prevention Group, Institut d’Investigació Biomèdica de Bellvitge, L’Hospitalet de Llobregat, Spain; 6 Department of Clinical Sciences, School of Medicine, Universitat de Barcelona, L'Hospitalet del Llobregat, Spain; 7 Environmental and Occupational Epidemiology Unit, Cancer Prevention and Research Institute, Florence, Italy; 8 Institute of Environmental Health, Medical University Vienna, Vienna, Austria; 9 Department of Environmental Epidemiology, Nofer Institute of Occupational Medicine, Lodz, Poland; 10 Tobacco Free Research Institute, The Digital Depot, Dublin, Ireland; 11 Office Français de Prevention du Tabagisme, Paris, France; 12 Tobacco Control Unit, Fondazione Istituto di Ricerca e Cura a Carattere Scientifico (IRCCS) Istituto Nazionale dei Tumori, Milan, Italy; 13 University of Minho, Portugal, Braga, Portugal; 14 Environmental Tobacco Smoke Research Laboratory, Tobacco Control Unit, Fondazione Istituto di Ricerca e Cura a Carattere Scientifico (IRCCS) Istituto Nazionale dei Tumori/Società Italiana di Medicina Generale (SIMG) Italian College General Practitioners, Milan, Italy; 15 Stop fajceniu, obcianske zdruzenie/Stop smoking - NGO, Bratislava, Slovakia; 16 Department of Experimental and Health Sciences, Universitat Pompeu Fabra, Barcelona, Spain; University of Montreal, Canada

## Abstract

**Background:**

Outdoor secondhand smoke (SHS) concentrations are usually lower than indoor concentrations, yet some studies have shown that outdoor SHS levels could be comparable to indoor levels under specific conditions. The main objectives of this study were to assess levels of SHS exposure in terraces and other outdoor areas of hospitality venues and to evaluate their potential displacement to adjacent indoor areas.

**Methods:**

Nicotine and respirable particles (PM2.5) were measured in outdoor and indoor areas of hospitality venues of 8 European countries. Hospitality venues of the study included night bars, restaurants and bars. The fieldwork was carried out between March 2009 and March 2011.

**Results:**

We gathered 170 nicotine and 142 PM2.5 measurements during the study. The median indoor SHS concentration was significantly higher in venues where smoking was allowed (nicotine 3.69 µg/m3, PM2.5: 120.51 µg/m3) than in those where smoking was banned (nicotine: 0.48 µg/m3, PM2.5: 36.90 µg/m3). The median outdoor nicotine concentration was higher in places where indoor smoking was banned (1.56 µg/m3) than in venues where smoking was allowed (0.31 µg/m3). Among the different types of outdoor areas, the highest median outdoor SHS levels (nicotine: 4.23 µg/m3, PM2.5: 43.64 µg/m3) were found in the semi-closed outdoor areas of venues where indoor smoking was banned.

**Conclusions:**

Banning indoor smoking seems to displace SHS exposure to adjacent outdoor areas. Furthermore, indoor settings where smoking is banned but which have a semi-closed outdoor area have higher levels of SHS than those with open outdoor areas, possibly indicating that SHS also drifts from outdoors to indoors. Current legislation restricting indoor SHS levels seems to be insufficient to protect hospitality workers – and patrons – from SHS exposure. Tobacco-free legislation should take these results into account and consider restrictions in the terraces of some hospitality venues to ensure effective protection.

## Introduction

Secondhand smoke (SHS) exposure causes premature mortality and morbidity, increasing the risk of numerous diseases such as lung cancer and coronary heart disease in non-smoking adults [1]. In addition, an increased risk for other conditions such as respiratory symptoms or low birth weight has been also shown in children. It is also important to notice that there is no safe level of SHS exposure. For this reason, smoke-free legislation have been widely developed and implemented during the last years. Despite the generalization of smoke-free workplaces [2], several European studies have shown extremely high levels of SHS exposure in hospitality venues in countries without complete smoking regulations, especially in some types of venue such as night clubs and musical bars [3], [4]. Furthermore, various studies [5]–[6] have shown that non-smoking hospitality workers have very high cotinine levels and a higher frequency of respiratory symptoms than other non-smokers. Studies evaluating recent smoke-free legislation have shown dramatic decreases in indoor SHS exposure levels [7]–[10] as well as significant decreases in respiratory symptoms in non-smoking hospitality workers [5]–[6].

A potential effect of indoor smoking restrictions is the displacement of smokers, and consequently of SHS, to outdoor areas. Consequently, SHS exposure in outdoor settings has become a growing public health concern [11]. Relocation of SHS outdoors might mitigate the results of indoor smoking bans, since both workers and clients would still be exposed. Furthermore, SHS from outdoor areas could drift inside, exposing people in supposedly protected areas to significant levels of SHS [12]. Several studies on outdoor SHS – also called outdoor tobacco smoke by some authors [13] - have recently been published. Some have measured particles with a diameter of 2.5 µm or less (PM2.5) in hospitality venues [13]–[15] while others have focused on the potential influence of outdoor SHS on the indoor entrances of public buildings [12], [16]. Most of these studies agree that the main factors that could influence outdoor exposure are wind conditions, the number of smokers and the physical characteristics of outdoor areas (potential covers or walls) [13], [14], [16], [17]. The relation between the degree of enclosurement and the SHS exposure has been assessed in some studies, with the preliminary results suggesting that the presence of overhead covers or walls might be associated with higher levels of SHS exposure than those found in open outdoor areas. However, none of these studies has measured SHS levels in terraces and other outdoor areas in hospitality venues by using nicotine, a specific environmental SHS marker. The main objective of this study was to assess the level of SHS exposure in terraces and other outdoor areas of hospitality venues of eight European countries by measuring nicotine and PM2.5 concentrations, and to evaluate their potential displacement to adjacent indoor areas.

## Methods

### Design and Population

We measured nicotine and PM2.5 measured in hospitality venues of major cities in the eight European countries involved in the IMPASHS (evaluation of the impact of smoke-free policies in Member States on exposure to second-hand smoke and tobacco consumption) project: Austria, France, Ireland, Italy, Poland, Portugal, Slovak Republic, and Spain.

The main objective of the study was to compare the indoor and outdoor SHS concentrations between venues where indoor smoking was allowed and venues where it was banned. For this reason, based on the average and standard deviations obtained in previous studies, we assessed the sample size needed in each group (indoor and outdoor). Regarding nicotine, to find an average standardized difference of 3 µg/m3 of indoor nicotine concentration with a statistical power of 80%, we needed 30 venues in each comparison group. For outdoor nicotine concentration, to find a difference of 1 µg/m3 we needed 23 venues in each group. Regarding PM2.5, to find a difference of 70 µg/m3 of indoor PM with a statistical power of 80%, we needed two groups with 31 venues each. For outdoor PM, to find a difference of 15 µg/m3 we needed 30 venues in each group. As all the groups used in the study included a minimum of 32 venues, the comparability between groups was therefore conveniently ensured.

The fieldwork was carried out between March 2009 and March 2011. We grouped hospitality venues in the study in three categories: night bars, restaurants, and bars. We defined night bars as any kind of musical bar open at night, restaurants as hospitality venues where food and drinks were served, and bars as hospitality venues where only drinks were served. We studied six venues (two of each type sampled in two different seasons) per country in summer and winter. In night bars, we took measurements after dinner, in restaurants either at lunch or dinner time, and in bars at any time. We selected the venues by convenience sampling based on the type of setting and smoking regulation. We used two selection criteria: 1) the absence of an open kitchen or other important sources of combustion in the venue, and 2) the presence of at least five people at the venue when the measurement was taken.

### Study Variables

We measured environmental nicotine and PM2.5 outdoors and indoors in the selected hospitality venues. The nicotine and PM2.5 measurements were carried out simultaneously.

We measured vapour phase nicotine using environmental tobacco smoke passive samplers, following Hammond’s validated method, as previously described [18]. Briefly, the sampler consisted of a 37-mm diameter plastic cassette containing a filter treated with sodium bisulphate. The samplers were attached to an air pump with a flow rate ranging from 2 to 3 l/min, and 30-min measurements were taken indoors and outdoors. The nicotine analysis was conducted at the Laboratory of the Public Health Agency of Barcelona by the gas chromatography/mass spectrometry method. The limit of quantification was 5 ng per filter. Samples with values under the limit of quantification were assigned half of this value. We estimated the time-weighted average nicotine concentration (µg/m3) by dividing the amount of extracted nicotine by the volume of air sampled (estimated flow rate multiplied by the total number of minutes the filter had been exposed).

We measured PM2.5 using either TSI SidePak AM510 Personal Aerosol Monitors, an Aerocet 531 monitor, or a Grimm Aerosol spectrometer. We adjusted all the measurements according to the calibration factor derived for each monitor in an experimental study [19]. In that study, all the monitors used in the IMPASHS project were calibrated against a BAM-1020 instrument that measured airborne particulate concentrations by using the principle of beta-ray attenuation. We downloaded the recorded measurements to a personal computer for analysis.

For each nicotine and PM2.5 measurement, we recorded the following data: the sample’s code, city, type of venue, date, starting and ending time, area (indoor/outdoor), smoking policy (smoking allowed/smoking banned), number of smokers, and type of outdoor area (open/semi-closed). An “open area” was defined as an outdoor area with no cover and no surrounding walls, while a semi-closed area was defined as an outdoor area with at least one wall or overhead cover. Finally, we recorded information on the sampling area, sampling volume and ventilation in each establishment to evaluate extreme or inconsistent values. We did not require approval from the ethics committee because the study did not involve interventions or measurements in humans but rather environmental measures.

### Statistical Analysis

Given the skewed distribution of PM2.5 and nicotine concentrations, we used medians and interquartile ranges (IQRs) to describe the data by area, type of venue, and season. We used the Wilcoxon and Mann-Whitney U-tests to compare medians according to the dependent or independent nature of the samples, respectively. In order to correct the potential problem of multiple comparisons, we used the Bonferroni correction, a conservative approach which sets the alpha value for each comparison equal to the fixed alpha value divided by the total number of comparisons. Analyses were performed using SPSS 18.0.

## Results

We gathered 170 nicotine samples and 142 PM2.5 samples during the study. The median indoor concentration was significantly higher in venues where smoking was allowed (nicotine: 3.69 µg/m^3^, PM2.5: 120.51 µg/m^3^) than in those where it was banned (nicotine: 0.48 µg/m^3^, PM2.5: 36.90 µg/m^3^). The outdoor nicotine concentration was significantly higher in places where indoor smoking was banned (1.56 µg/m^3^, IQR: 0.22–5.82) than in those where it was allowed (0.31 µg/m^3^, IQR: 0.14–0.66) (Table 1).

**Table 1:** Nicotine and PM2.5 concentrations (µg/m^3^) by area and smoking regulation (paired samples). IMPASHS study, 2009–2011.

		Indoor area		Outdoor area		p- value a
		n	Median (IQR)	n	Median (IQR)	
**Nicotine**						
Indoor smoking allowed		46	3.69 (0.42–15.78)	46	0.31 (0.14–0.66)	<0.01**
Indoor smoking banned		39	0.48 (0.22–3.01)	39	1.56 (0.22–5.82)	0.13
p- value b			<0.01**		<0.01**	
**PM2.5**						
Indoor smoking allowed		42	120.51 (31.20–212.16)	42	29.61 (18.72–42.24)	<0.01**
Indoor smoking banned		32	36.90 (19.75–85.18)	32	36.10 (16.24–63.91)	0.13
p- value b			0.02*		0.35	

a Wilcoxon test for comparison of medians from indoor/outdoor areas.

b Mann-Whitney U-test for comparison of medians from areas where smoking was allowed/banned.

* p<0.05.

** p<0.016 (significance level of 0.05 adjusted for Bonferroni correction for 4 comparisons).

Regardless of the type of venue, indoor nicotine and PM2.5 concentrations were consistently higher in places where smoking was allowed than in those where it was not allowed (Table 2). Where smoking was allowed, we found the highest indoor nicotine concentration in restaurants (8.52 µg/m^3^, IQR: 0.70–19.62). Where indoor smoking was banned, we found the highest outdoor nicotine concentration in night bars (2.85 µg/m^3^, IQR: 0.88–8.81).

**Table 2:** Nicotine and PM2.5 concentrations (µg/m^3^) by type of venue, area and smoking regulation. IMPASHS study, 2009–2011.

	Indoor area		Outdoor area		
	n	Median (IQR)	n	Median (IQR)	p-value^a^
**Nicotine**					
**Night bar**					
Smoking allowed	16	3.88 (0.48–16.91)	16	0.43 (0.19–1.50)	0.03*
Smoking banned	14	0.91 (0.40–3.01)	14	2.85 (0.88–8.81)	0.24
p- value b		0.18		0.02*	
**Restaurant**					
Smoking allowed	15	8.52 (0.70–19.62)	15	0.29 (0.12–0.47)	<0.01**
Smoking banned	13	0.79 (0.22–3.40)	13	0.66 (0.18–4.23)	0.92
p- value b		0.05*		0.10	
**Bar**					
Smoking allowed	15	1.92 (0.20–10.14)	15	0.29 (0.11–0.74)	0.02*
Smoking banned	12	0.43 (0.16–1.13)	12	1.25 (0.18–6.54)	0.18
p- value b		0.10		0.11	
**PM2.5**					
**Night bar**					
Smoking allowed	15	119.08 (25.48–269.00)	15	26.28 (18.72–65.79)	0.01*
Smoking banned	10	59.97 (19.89–144.02)	10	51.29 (32.50–64.77)	0.09
p-value b		0.53		0.34	
**Restaurant**					
Smoking allowed	13	121.93 (44.20–149.63)	13	31.87 (19.89–42.33)	0.04*
Smoking banned	11	68.85 (25.00–92.56)	11	22.30 (13.26–45.43)	0.16
p- value b		0.28		0.69	
**Bar**					
Smoking allowed	14	140.50 (31.62–212.16)	14	27.76 (12.61–29.64)	<0.01**
Smoking banned	11	28.56 (16.00–40.80)	11	29.60 (17.85–64.48)	0.45
p- value b		0.02*		0.32	

a Wilcoxon test for comparison of medians from indoor/outdoor areas.

b Mann-Whitney U-test for comparison of medians from areas where smoking was allowed/banned.

* p<0.05.

** p<0.004 (significance level of 0.05 adjusted for Bonferroni correction for 12 comparisons).

Indoor nicotine and PM concentrations in venues where smoking was allowed were significantly higher in winter (nicotine: 10.88 µg/m^3^, PM2.5: 149.63 µg/m^3^) than in summer (nicotine: 0.74 µg/m^3^, PM2.5: 59.16 µg/m^3^), indicating a seasonal pattern (Table 3). The median outdoor nicotine concentration in winter was higher (3.47 µg/m^3^, IQR: 0.59–8.05) in venues where indoor smoking was banned than in those where indoor smoking was allowed (0.50 µg/m^3^ (IQR: <Limit of quantification –1.67).

**Table 3:** Nicotine and PM2.5 concentrations (µg/m^3^) by season, area and smoking regulation (paired samples). IMPASHS study, 2009–2011.

	Indoor area		Outdoor area		
	n	Median (IQR)	n	Median (IQR)	p-value^a^
**Nicotine**					
**Summer**					
Smoking allowed	24	0.74 (0.21–4.94)	24	0.26 (0.15–0.46)	<0.01**
Smoking banned	17	0.45 (0.22–1.85)	17	0.88 (0.13–2.87)	0.48
p- value b		0.20		0.14	
**Winter**					
Smoking allowed	22	10.88 (3.28–21.61)	22	0.50 (<LQ –1.67)	<0.01**
Smoking banned	22	0.84 (0.28–3.51)	22	3.47 (0.59–8.05)	0.20
p- value b		<0.01**		0.03*	
**PM2.5**					
**Summer**					
Smoking allowed	23	59.16 (21.93–164.26)	23	29.58 (13.95–42.33)	<0.01**
Smoking banned	16	23.46 (10.70–63.12)	16	18.93 (8.70–61.82)	0.84
p- value b		0.12		0.98	
**Winter**					
Smoking allowed	19	149.63 (112.20–269.00)	19	29.64 (21.84–42.24)	<0.01**
Smoking banned	16	73.32 (31.69–139.37)	16	39.89 (29.00–85.22)	0.08
p- value b		0.03*		0.18	

a Wilcoxon test for comparison of medians from indoor/outdoor areas.

b Mann-Whitney U-test for comparison of medians from areas where smoking was allowed/banned.

LQ: Limit of quantification.

* p<0.05.

** p<0.006 (significance level of 0.05 adjusted for Bonferroni correction for 8 comparisons).

Among outdoor areas, we found the highest outdoor nicotine and PM2.5 levels in the semi-closed outdoor areas of venues where indoor smoking was banned (median nicotine concentration 4.23 µg/m^3^, median PM2.5 concentration: 43.64 µg/m^3^) (Table 4). The median indoor nicotine concentration increased with the number of smokers present in semi-closed outdoor areas (0 smokers: 0.30 µg/m^3^ [IQR:0.19–2.87], 1–8 smokers: 0.02 [IQR: 0.63–7.25], >8 smokers: 4.23 [0.19–2.87]). We observed no differences in open outdoor areas, although outdoor nicotine concentration in semi-closed areas tended to increase according to the number of smokers (data not shown).

**Table 4:** Nicotine and PM2.5 concentrations (µg/m^3^) by type of outdoor area (paired samples). IMPASHS study, 2009–2010.

	Indoor area		Outdoor area		
	n	Median (IQR)	n	Median (IQR)	p-value^a^
**Nicotine**					
**Open area** c					
Smoking allowed	32	4.03 (0.42–19.62)	32	0.41 (0.13–0.60)	<0.01**
Smoking banned	19	0.40 (0.10–1.05)	19	0.58 (0.13–1.66)	0.60
p- value b		<0.01**		0.39	
**Semi-closed** d					
Smoking allowed	14	2.71 (0.42–9.42)	14	0.19 (0.14–1.26)	0.06*
Smoking banned	20	1.44 (0.32–4.61)	20	4.23 (1.56–8.43)	0.15
p- value b		0.55		0.01*	
**PM2.5**					
**Open area** c					
Smoking allowed	28	94.51 (31.41–211.33)	28	27.76 (18.46–33.60)	<0.01**
Smoking banned	16	32.31 (19.75–75.38)	16	25.45 (13.06–43.12)	0.17
p- value b		0.02*		0.99	
**Semi-closed** d					
Smoking allowed	14	132.01 (21.93–234.60)	14	38.24 (19.89–65.79)	0.04*
Smoking banned	16	59.97 (17.95–141.39)	16	43.64 (23.73–109.48)	0.47
p- value b		0.31		0.49	

a Wilcoxon test for comparison of medians from indoor/outdoor areas.

b Mann-Whitney U-test for comparison of medians from areas where smoking was allowed/banned.

c Open area was defined as an outdoor area with no roof and no surrounding walls.

d Semi-closed was defined as an outdoor area with at least one wall or roof.

* p<0.05.

** p<0.006 (significance level of 0.05 adjusted for Bonferroni correction for 8 comparisons).

## Discussion

The results of our study show that SHS levels in terraces and other outdoor areas of hospitality venues where indoor smoking is banned are significantly higher than in those of hospitality venues where smoking is allowed, indicating displacement of SHS exposure to adjacent outdoor areas. Furthermore, outdoor SHS levels are much higher in semi-closed terraces (defined as those having at least one wall or roof) than in open outdoor areas. Finally, indoor settings where smoking is banned but which have a semi-closed outdoor area have higher levels of SHS than those with open outdoor areas, suggesting that SHS may also drift from outdoors to indoors, exposing patrons or workers inside the venue to SHS from outdoors.

### Relation to Other Studies

Our finding that outdoor nicotine concentration was significantly higher in venues where indoor smoking was banned suggests that indoor smoking bans may increase SHS in outdoor areas. This finding is consistent with the results of a previous study carried out in bars and restaurants in Georgia [11], reporting that the salivary cotinine levels of non-smokers in outdoor areas of çbars and restaurants where indoor smoking was banned significantly increased from pre-test to post-test in people exposed to the outdoor areas of bars and restaurants compared with a control group.

Equally, our finding that when indoor smoking is banned, SHS levels in semi-closed outdoor areas are much higher than in open patios or outdoor areas is consistent with the results of an Australian study that measured PM2.5 in different types of outdoor dining areas. The authors found that being situated under an overhead cover increased average SHS exposure by around 50% [15]. Similarly, another study that measured SHS levels in the entrances of public buildings showed that SHS levels in “quasi-outdoor” entrances – defined as outdoor entrances with an overhead cover and/or side walls – were higher than those in uncovered main entrances. It is also important to notice that, according to the data obtained in our study, the SHS levels in semiclosed outdoor areas where indoor smoking is banned may be even higher than indoor SHS levels where indoor smoking is permitted. This result shows that banning indoor smoking may not be enough to protect people from the SHS exposure. Therefore, a smoking ban in outdoor areas with overhead cover or walls would be necessary in order to protect customers and workers from SHS exposure.

Our finding of higher indoor SHS levels in semi-closed outdoor areas provides further evidence for the hypothesis that outdoor SHS drifts to adjacent indoor areas, as previously proposed by Klepeis [13]. This hypothesis was also supported by the study of Sureda et al. [16], where the PM2.5 concentrations obtained in the main outdoor entrances of public buildings were reported to be similar to those obtained simultaneously in adjacent indoor halls, and at the same time higher than control points outdoors and indoors.

Finally, although not significant, we found that the outdoor nicotine concentration in semi-closed areas tended to increase according to the number of smokers. A similar result was observed in public buildings in Australia, where the median outdoor PM2.5 level increased from 8.0 µg/m3 with no lit cigarettes to 19.5 µg/m^3^ with more than 5 lit cigarettes [12]. Another Australian study carried out in “alfresco areas” reported a dose response increase in mean PM2.5 concentrations for none, one and two or more smokers (with 3.98, 10.59 and 17.00 µg/m3 respectively) [20]. A study performed in 2007 reported that in outdoor restaurant patios, more than 8 cigarettes smoked sequentially could cause an incremental 24-hr particle exposure greater than a threshold level of 35 mg/m^3^ for a person within 0.5 m of the smokers [13].

### Strengths and Limitations

A potential limitation of our study is that we used a convenience sampling of hospitality venues, which could affect the study’s external validity. However, we attempted to minimize the potential selection bias by stratifying the selection of the venues by the main potential confounders such as the type of venue, smoking regulation and geographical area. In contrast, we did not account for some of the factors affecting outdoor SHS identified in previous studies, such as the distance and position of smokers relative to the sampling equipment and wind speed or direction. We neither recorded other variables that could be affecting the SHS concentrations such as the movement of people between indoor and outdoor areas. Traffic of people from outdoors to indoors could favor the drift of tobacco smoke from outdoors to indoors, according to the number of people moving and also to the time the doors will remain open. While these variable are difficult to be recorded, future studies should contemplate to include this type of information. However, we recorded two of the main factors affecting outdoor SHS exposure: the type of outdoor area (semi-closed or open) and the number of smokers. Finally, the limited duration of the measurements (30 minutes) might not reflect typical exposure. Nevertheless, our methods constitute a reliable approach to “real exposure”, avoiding the underestimation that may be associated with passive methods that take measurements for several days, including the hours while the venues are closed.

To our knowledge this is the first study simultaneously measuring two environmental SHS markers in outdoor and indoor areas of hospitality venues in Europe. Importantly, we measured a specific air marker of SHS in outdoor areas of hospitality venues, while most previous studies only measured PM2.5 (13 - 15], which could be influenced by other combustion sources, such as diesel cars or cooking sources. Furthermore, our study includes measurements in summer and winter, providing extra information on how seasonality affects outdoor smoking.

### Implications for Legislation

Outdoor smoking bans have been extensively discussed in the last few years. Some arguments against these bans are the absence of evidence on outdoor SHS levels and the potential health effects of outdoor exposure [21], as well as the fact that outdoor SHS dissipates further than indoor SHS [13]. Authors in favor of these bans argue that there is no safe level of SHS exposure [22], that there is evidence that outdoor exposure can be as high as indoor smoking environments under certain conditions [13], and that outdoor bans would reduce smoking being modeled to children as normal behavior [23]. Support for smoke-free outdoor public places among the general population appears to be increasing, as shown by several surveys [24]. Respondents’ reported reasons for support were litter control, to establish positive smoke-free role models for youth, to reduce youth opportunities to smoke, and to avoid SHS exposure.

According to the Framework Convention on Tobacco Control guidelines, “outdoor or quasi-outdoor public places where appropriate (including all outdoor public places where tobacco smoke is a health hazard) should be 100% smoke-free” [25]. Although more evidence may be needed to determine whether a ban on outdoor smoking is required because of a health risk (and if so, in which settings or places), some restrictions such as limits on outdoor areas close to certain entrances or smoking bans in selected semi-closed outdoor areas seem reasonable. In 2005, Repace declared in one of his reports that “It makes sense to post signs warning smokers not to smoke closer than about 20 feet from building entrances” [17]. Restrictions in semi-closed areas have already been implemented in several cities such as Ontario, where smoking is banned in “outdoor public places or workplaces with roofs, overhangs or awnings” [26].

### Conclusions

Overall, this study shows that SHS levels in the semi-closed outdoor areas of hospitality venues might be high, possibly indicating an unacceptable risk, especially for hospitality workers. Current legislation restricting indoor SHS levels seems to be insufficient to protect hospitality workers – and patrons – from SHS exposure. Although further research may be needed on this topic, tobacco-free legislation should take these results into account and consider restrictions in the terraces of some hospitality venues to ensure effective protection.

## References

[1] U.S.Department of Health and Human Services (2006) The Health Consequences of Involuntary Exposure to Tobacco Smoke: A Report of the Surgeon General. Washington, DC. 709 p. 20669524

[2] IARC Handbooks of Cancer Prevention (2009) Evaluating the effectiveness of smoke-free policies. [13]. Lyon.

[3] Nebot M, Lopez MJ, Ariza C, Perez-Rios M, Fu M, et al (2009) Impact of the Spanish smoking law on exposure to secondhand smoke in offices and hospitality venues: before-and-after study. Environ Health Perspect 117: 344–347. 19337506 10.1289/ehp.11845 PMC2661901

[4] Lopez MJ, Nebot M, Albertini M, Birkui P, Centrich F, et al (2008) Secondhand smoke exposure in hospitality venues in Europe. Environ Health Perspect 116: 1469–1472. 19057698 10.1289/ehp.11374 PMC2592265

[5] Fernandez E, Fu M, Pascual JA, Lopez MJ, Perez-Rios M, et al (2009) Impact of the Spanish smoking law on exposure to second-hand smoke and respiratory health in hospitality workers: a cohort study. PLoS One 4: e4244. 19165321 10.1371/journal.pone.0004244 PMC2621339

[6] Allwright S, Paul G, Greiner B, Mullally BJ, Pursell L, et al (2005) Legislation for smoke-free workplaces and health of bar workers in Ireland: before and after study. BMJ 331: 1117. 16230313 10.1136/bmj.38636.499225.55 PMC1283274

[7] Callinan JE, Clarke A, Doherty K, Kelleher C (2010) Legislative smoking bans for reducing secondhand smoke exposure, smoking prevalence and tobacco consumption. Cochrane Database Syst RevCD005992. 10.1002/14651858.CD005992.pub2 20393945

[8] Apsley A, Semple S (2012) Secondhand smoke levels in Scottish bars 5 years on from the introduction of smoke-free legislation Tob Control: In press. 10.1136/tobaccocontrol-2011-050107 22016506

[9] Lopez MJ, Nebot M, Schiaffino A, Perez-Rios M, Fu M, et al (2012) Two-year impact of the Spanish smoking law on exposure to secondhand smoke: evidence of the failure of the 'Spanish model' (2011) Tob Control. 21: 407–411. 10.1136/tc.2010.042275 21659449

[10] Gorini G, Moshammer H, Sbrogio L, Gasparrini A, Nebot M, et al (2008) Italy and Austria before and after study: second-hand smoke exposure in hospitality premises before and after 2 years from the introduction of the Italian smoking ban. Indoor Air 18: 328–334. 18429994 10.1111/j.1600-0668.2008.00534.x

[11] Hall JC, Bernert JT, Hall DB, St Helen G, Kudon LH, et al (2009) Assessment of exposure to secondhand smoke at outdoor bars and family restaurants in Athens, Georgia, using salivary cotinine. J Occup Environ Hyg 6: 698–704. 19757294 10.1080/15459620903249893

[12] Kaufman P, Zhang B, Bondy SJ, Klepeis N, Ferrence R (2011) Not just 'a few wisps': real-time measurement of tobacco smoke at entrances to office buildings. Tob Control 20: 212–218. 21177666 10.1136/tc.2010.041277

[13] Klepeis NE, Ott WR, Switzer P (2007) Real-time measurement of outdoor tobacco smoke particles. J Air Waste Manag Assoc 57: 522–534. 17518219 10.3155/1047-3289.57.5.522

[14] Brennan E, Cameron M, Warne C, Durkin S, Borland R, et al (2010) Secondhand smoke drift: examining the influence of indoor smoking bans on indoor and outdoor air quality at pubs and bars. Nicotine Tob Res 12: 271–277. 20097839 10.1093/ntr/ntp204

[15] Cameron M, Brennan E, Durkin S, Borland R, Travers MJ, et al (2010) Secondhand smoke exposure (PM2.5) in outdoor dining areas and its correlates. Tob Control 19: 19–23. 19850553 10.1136/tc.2009.030544

[16] Sureda X, Martinez-Sanchez JM, Lopez MJ, Fu M, Aguero F, et al (2011) Secondhand smoke levels in public building main entrances: outdoor and indoor PM2.5 assessment. Tob Control [Epub ahead of print]. 10.1136/tobaccocontrol-2011-050040 21964181

[17] Repace J (2005) Measurements of outdoor air pollution from secondhand smoke on the UMBC campus. Available at: http://www.repace.com/pdf/outdoorair.pdf. Accessed November 2011..

[18] Hammond SK (1993) Evaluating exposure to environmental tobacco smoke. In: Sampling and Analysis of Airborne Pollutants (Winegar ED LH, ed). Florida:CRC Press;319–337.

[19] Ruprecht AA, De Marco C, Boffi R, Mazza R, Lopez MJ, et al (2011) Mass calibration and Relative Humidity compensation requirements for optical portable particulate matter monitors : the IMPASHS (Impact of smoke-free policies in EU Member States) WP2 preliminary results. Epidemiology 22: s206.

[20] Stafford J, Daube M, Franklin P (2010) Second hand smoke in alfresco areas. Health Promot J Austr 21: 99–105. 20701558 10.1071/he10099

[21] Chapman S (2008) Should smoking in outside public spaces be banned? No. BMJ 337: a2804. 10.1136/bmj.a2804 19074219

[22] World Health Organization (2000) Air quality guidelines, 2nd ed. WHO Regional Publications, European Series, No.91.WHO, Regional Office for Europe. 11372513

[23] Thomson G, Wilson N, Edwards R, Woodward A (2008) Should smoking in outside public spaces be banned? Yes. BMJ 337: a2806. 19074220 10.1136/bmj.a2806

[24] Thomson G, Wilson N, Edwards R (2009) At the frontier of tobacco control: a brief review of public attitudes toward smoke-free outdoor places. Nicotine Tob Res 11: 584–590. 19359392 10.1093/ntr/ntp046

[25] World Health Organization (WHO) (2009) WHO Report of the Global Tobacco Epidemic. Implementing smoke-free environments. Geneve.

[26] Kennedy RD, Elton-Marshall T, Mutti S, Dubray J, Fong GT (2010) Understanding the impact of the Smoke-Free Ontario Act on hospitality establishments' outdoor environments: a survey of restaurants and bars. Tob Control 19: 165–167. 20378593 10.1136/tc.2009.031872

